# Jogging and weight training associated with increased high-density lipoprotein cholesterol levels in Taiwanese adults

**DOI:** 10.1080/15502783.2022.2145232

**Published:** 2022-11-29

**Authors:** Chien-Chang Ho, Oswald Ndi Nfor, Yun-Tsung Chen, Chi-Fang Lin, Wen-Yu Lu, Min-Chen Wu, Chuan-Chao Lin, Yung-Po Liaw

**Affiliations:** a Department of Physical Education, Fu Jen Catholic University, New Taipei, Taiwan; b Research and Development Center for Physical Education, Health, and Information Technology, College of Education, Fu Jen Catholic University, New Taipei, Taiwan; c Sports Medicine Center, Fu Jen Catholic University Hospital, New Taipei, Taiwan; d Department of Public Health and Institute of Public Health, Chung Shan Medical University, Taichung, Taiwan; e Department of Physical Education and Sport Sciences, National Taiwan Normal University, Taipei, Taiwan; f Office of Physical Education, Chung Yuan Christian University, Taoyuan, Taiwan; g Department of Physical Medicine and Rehabilitation, Chung Shan Medical University Hospital, Taichung, Taiwan; h School of Medicine, Chung Shan Medical University, Taichung, Taiwan; i Department of Medical Imaging, Chung Shan Medical University Hospital, Taichung, Taiwan; j Institute of Medicine, Chung Shan Medical University, Taichung, Taiwan

**Keywords:** Physical activity, high-density lipoprotein, Taiwan Biobank

## Abstract

**Background:**

Although previous studies have shown that aerobic and resistance exercise increase high-density lipoprotein cholesterol (HDL-C) levels, the optimal type of exercise has not been determined. Therefore, the purpose of this study was to investigate the association of jogging (a type of aerobic exercise) and weight training (a type of resistance exercise) with HDL-C levels in Taiwanese adults.

**Methods:**

The data used in this cross-sectional study were obtained from the Taiwan Biobank (TWB), which is a national health resource that contains the genetic information of Taiwanese volunteers aged 30–70 years. A total of 75,635 subjects (47,881 women and 27,754 men) were included in this study. The subjects were divided into four groups: jogging (*n* = 2,278), weight training (*n* = 522), mixed exercise (*n* = 519), and no exercise (*n* = 72,316). The TWB data were collected through questionnaires (e.g. basic characteristics, lifestyle factors, and disease history), biochemical tests, and anthropometric measurements.

**Results:**

Compared with no exercise, jogging, weight training, and mixed exercise were all associated with higher HDL-C levels (β = 2.5470, 2.6249, and 3.2117, respectively). As seen, the β value was highest for the mixed exercise group, followed by weight training and then jogging (*p* for trend <0.0001).

**Conclusions:**

In the current study, jogging and weight training were individually associated with higher levels of HDL-C. Engaging in both activities was associated with much higher levels of HDL-C. Our findings suggest that regular jogging and weight training might play an important role in increasing HDL-C levels.

## 1. Background

Cardiovascular disease (CVD) is one of the leading causes of death in Taiwan [1]. It accounts for approximately 30% of deaths worldwide [2,3]. High-density lipoprotein (HDL) is a well-established, independent predictor of cardiovascular disease [4–6]. However, its causal role in atherosclerotic cardiovascular disease is currently being disputed [4,5,7,8]. Nonetheless, this does not challenge the robustness of HDL concentration for predicting and stratifying cardiovascular diseases [4,5]. According to the United States National Cholesterol Education Program Adult Treatment Panel III guidelines, an HDL cholesterol (HDL-C) level of ≥60 mg/dL is a “negative” risk (protective) factor and <40 mg/dL is a high-risk factor [9].

Variations in HDL-C levels are caused by a variety of factors [10]. Family and twin studies have demonstrated that genetic factors influence 40–60% of HDL-C [11,12]. Non-genetic factors such as smoking and obesity greatly reduce HDL-C levels and have negative impacts on cardiovascular health [13]. However, exercise training mitigates the negative effects by increasing HDL-C levels [14–17]. The HDL-C-increasing role of exercise could, in part, be related to its effect on enzymes involved in cholesterol transport and metabolism. For example, exercise promotes the formation of HDL-C by increasing the expression of ATP-binding cassette transporter A-1 [18], which is an important protein in reverse cholesterol transport [19,20].

Based on exercise modalities, the relationship of aerobic and resistance exercise with HDL-C, other lipoproteins, and cardiovascular risk factors has been extensively explored in many randomized controlled trials (RCTs) and meta-analyses of RCTs [14,21–33]. The lipoprotein that is highly associated with aerobic exercise is HDL [34]. However, a combination of aerobic and resistance exercise is more beneficial for achieving cardiorespiratory fitness and losing weight and fat than aerobic or resistance exercise alone [14,35]. Only a few studies have examined the joint effects of aerobic and resistance exercise on the aforementioned factors [14,15,29,36]. Whatever the case may be, the results have been contentious [31].

RCTs, observational studies, and meta-analyses found that aerobic exercise and resistance training were associated with higher levels of HDL [14,16,21,22,28,37–40]. Nonetheless, some studies have found that doing these activities did not result in significant changes in HDL-C levels [23,25,26,30,41,42]. In other studies, a combination of aerobic exercise and resistance training was associated with increased HDL levels among overweight and obese men and women [14–16]. In other studies, however, combining both exercise modalities had no significant effect on HDL [43,44].

Most of the studies on HDL and aerobic or resistance exercise are RCTs or meta-analyses with a limited number of participants. While these activities have been extensively examined, little has been studied regarding how specific types of aerobic or resistance exercise might affect HDL. Jogging, a common type of aerobic exercise, involves running slowly or leisurely and can be performed by almost everyone since it requires no special facilities or skills [35,45,46]. Weight training is a common type of resistance exercise that involves lifting weights with or without joint movements [47,48]. Jogging and running have been reported to improve HDL levels [45,49]. However, in a study focusing on the role of weight training and jogging on HDL and other metabolic risk factors, both activities were associated with decreased HDL levels, but the decrease was significant only in the jogging group [35]. The study included only 26 women, and the authors recommended further research with larger sample sizes. It is, therefore, imperative to conduct research with larger sample sizes to gain a deeper understanding of how jogging and resistance training affect HDL. In this observational study, we examined the association between jogging, weight training, and the combination of both with HDL-C levels in Taiwanese adults.

## 2. Methods

### 2.1. Data source

Data were obtained from the Taiwan Biobank (TWB), a national health resource that contains the genetic information of Taiwanese volunteers collected at recruitment centers from 2008 to 2020. The TWB project has been previously described [12]. Detailed information about this project can be found at the biobank’s official website (https://taiwanview.twbiobank.org.tw/index). In brief, the TWB project aims to collect genetic information from over 200,000 Taiwanese aged 30–70 years by 2027. Data collection at designated recruitment centers is done using standardized procedures. All the volunteers usually provide written informed consent before data collection. The collection procedure entails undergoing serial physical examinations (height, weight, waist circumference [WC], hip circumference, body fat percentage, systolic blood pressure [SBP], and diastolic blood pressure [DBP]), biospecimen collection, and self-response to the TWB questionnaire (basic characteristics, lifestyle factors, and disease history).

## 3. Study subjects and their characteristics

The following variables were considered in the current study: age, sex, education, marital status, fasting blood glucose, creatinine, uric acid, total cholesterol (TC), triglycerides, low-density lipoprotein cholesterol (LDL-C), HDL-C, aspartate aminotransferase (AST), alanine aminotransferase (ALT), SBP, DBP, body mass index (BMI), WC, waist–hip ratio (WHR), body fat percentage, lifestyle factors (physical activity, cigarette smoking, alcohol consumption, and betel nut chewing), and relevant disease history (diabetes, hypertension, hyperlipidemia, and stroke).

Regular exercise was defined based on the weekly frequency of engaging in jogging and weight training (at least 3 days per week) and the duration of each exercise session (at least 30 min). Subjects were categorized into several physical activity groups including no exercise (did not engage in regular exercise), jogging (jogged three times or more per week and ≥30 min per session), weight training (engaged in weight training three times or more per week and ≥30 min per session), and mixed exercise (engaged both jogging and weight training times or more times per week and ≥30 min).

The total study sample size included 75,635 subjects, comprising 2,278, 522, 519, and 72,316 individuals in the jogging, weight training, mixed exercise, and no exercise group, respectively. Genetic and phenotypic data collected from these subjects between 2008 and 2015 were available in TWB. All investigations in this study were conducted in accordance with the Declaration of Helsinki. This study was approved by the Institutional Review Board of Chung Shan Medical University Hospital (CS1-20009).

## 4. Statistical analysis

Data management and statistical analyses were performed using SAS 9.4 (SAS Institute, Cary, NC, USA). A one-way analysis of variance (ANOVA) was used to compare the means ± standard deviation of HDL-C among the exercise groups. The chi-square test was used to compare the differences in categorical variables among the exercise groups. The categorical variables were presented as *n* (%), where *n* represents the number of individuals. Multivariate linear regression models were used to determine the association of the exercise types with HDL, and the results were presented as *β* coefficients. For all the analyses, a *p*-value of <0.05 was set as the statistically significant threshold.

## 5. Results

Overall, 75,635 subjects (47,881 women and 27,754 men) aged 30–70 years were included in the current study. Table 1 presents their characteristics stratified by exercise type. The exercise groups were significantly different with respect to HDL, sex, age, creatinine, uric acid, triglycerides, LDL, BMI, WHR, alcohol drinking, cigarette smoking, betel nut chewing, WC, body fat percentage, fasting blood glucose, SBP, DBP, education, marital status, diabetes, hypertension, hyperlipidemia, and stroke.

**Table 1.** Characteristics of the study subjects stratified by exercise type.

Variable	No exercise (*n* = 72,316)	Exercise type			
		Jogging (*n* = 2278)	Weight training (*n* = 522)	Mixed (*n* = 519)	*p*-Value
**HDL-C (mg/dL)**	53.787 ± 0.049	55.349 ± 0.287	55.349 ± 0.589	54.536 ± 0.621	<0.0001
**Sex**					<0.0001
Female	46,865 (64.81)	672 (29.50)	214 (41.00)	130 (25.05)	
Male	25,451 (35.19)	1606 (70.50)	308 (59.00)	389 (74.95)	
**Age (years)**					<0.0001
30-45	35,707 (49.38)	1215 (53.34)	370 (70.88)	401 (77.26)	
46-60	28,031 (38.76)	870 (38.19)	122 (23.37)	94 (18.11)	
≥61	8578 (11.86)	193 (8.47)	30 (5.75)	24 (4.62)	
**Creatinine (mg/dL)**					0.0427
<1.4	71,969 (99.52)	2273 (99.78)	522 (100.00)	519 (100.00)	
≥1.4	347 (0.48)	5 (0.22)	0 (0.00)	0 (0.00)	
**Uric acid (mg/dL)**					<0.0001
Male ≤7; female ≤6	58,580 (81.01)	1697 (74.50)	421 (80.65)	369 (71.10)	
Male >7; female >6	13,736 (18.99)	581 (25.50)	101 (19.35)	150 (28.90)	
**Total cholesterol (mg/dL)**					0.0775
<200	42,772 (59.15)	1362 (59.79)	332 (63.60)	324 (62.43)	
≥200	29,544 (40.85)	916 (40.21)	190 (36.40)	195 (37.57)	
**Triglycerides (mg/dL)**					<0.0001
<150	56,468 (78.09)	1893 (83.10)	436 (83.52)	421 (81.12)	
≥150	15,848 (21.91)	385 (16.90)	86 (16.48)	98 (18.88)	
**LDL-C (mg/dL)**					0.0416
<130	45,750 (63.26)	1506 (66.11)	334 (63.98)	336 (64.74)	
≥130	26,566 (36.74)	772 (33.89)	188 (36.02)	183 (35.26)	
**BMI (kg/m^2^)**					<0.0001
Normal (18.5 ≤ BMI < 24)	34,548 (47.77)	1129 (49.56)	269 (51.53)	203 (39.11)	
Underweight (BMI <18.5)	2774 (3.84)	57 (2.50)	8 (1.53)	5 (0.96)	
Overweight (24 ≤ BMI < 27)	18,772 (25.96)	697 (30.60)	141 (27.01)	188 (36.22)	
Obese (BMI ≥27)	16,222 (22.43)	395 (17.34)	104 (19.92)	123 (23.70)	
**WHR**					<0.0001
Male <0.9; female <0.8	24,987 (34.55)	1323 (58.08)	281 (53.83)	329 (63.39)	
Male ≥0.9; female ≥0.8	47,329 (65.45)	955 (41.92)	241 (46.17)	190 (36.61)	
**Alcohol drinking**					<0.0001
Never	66,480 (91.93)	1973 (86.61)	467 (89.46)	448 (86.32)	
Quit	1677 (2.32)	75 (3.29)	13 (2.49)	15 (2.89)	
Current	4159 (5.75)	230 (10.10)	42 (8.05)	56 (10.79)	
**Cigarette smoking**					<0.0001
Never	57,650 (79.72)	1587 (69.67)	382 (73.18)	371 (71.48)	
Quit	6425 (8.88)	441 (19.36)	65 (12.45)	85 (16.38)	
Current	8241 (11.40)	250 (10.97)	75 (14.37)	63 (12.14)	
**Betel nut chewing**					0.0001
Never	67,722 (93.65)	2086 (91.57)	485 (92.91)	493 (94.99)	
Quit	4123 (5.70)	183 (8.03)	35 (6.70)	24 (4.62)	
Current	471 (0.65)	9 (0.40)	2 (0.38)	2 (0.39)	
**AST (U/L)**					0.0575
<40	68,518 (94.75)	2136 (93.77)	487 (93.30)	486 (93.64)	
≥40	3798 (5.25)	142 (6.23)	35 (6.70)	33 (6.36)	
**ALT** (**U/L)**					0.1975
<40	63,517 (87.83)	2026 (88.94)	467 (89.46)	449 (86.51)	
≥40	8799 (12.17)	252 (11.06)	55 (10.54)	70 (13.49)	
**Waist circumference (m)**					<0.0001
Male <90; female <80	37,846 (52.33)	1599 (70.19)	342 (65.52)	351 (67.63)	
Male ≥90; female ≥80	34,470 (47.67)	679 (29.81)	180 (34.48)	168 (32.37)	
**Body fat (%)**					<0.0001
Male <25; female <30	34,364 (47.52)	1585 (69.58)	311 (59.58)	338 (65.13)	
Male ≥25; female ≥30	37,952 (52.48)	693 (30.42)	211 (40.42)	181 (34.87)	
**Diabetes**					<0.0001
No	67,879 (93.86)	2195 (96.36)	511 (97.89)	511 (98.46)	
Yes	4437 (6.14)	83 (3.64)	11 (2.11)	8 (1.54)	
**Fasting blood glucose (mg/dL)**					<0.0001
<126	69,339 (95.97)	2233 (98.02)	515 (98.66)	514 (99.04)	
≥126	2917 (4.03)	45 (1.98)	7 (1.34)	5 (0.96)	
**Hypertension**					<0.0001
No	58,430 (80.80)	1816 (79.72)	460 (88.12)	457 (88.05)	
Yes	13,886 (19.20)	462 (20.28)	62 (11.88)	62 (11.95)	
**SBP (mmHg)**					<0.0001
<120	47,721 (59.08)	1149 (50.44)	309 (59.20)	271 (52.22)	
120–139	21,347 (29.52)	852 (37.40)	183 (35.06)	202 (38.92)	
≥140	8248 (11.41)	277 (12.16)	30 (5.75)	46 (8.86)	
**DBP (mmHg)**					<0.0001
<80	53,385 (73.82)	1543 (62.73)	421 (80.65)	394 (75.92)	
80–89	13,117 (18.14)	504 (22.12)	71 (13.60)	98 (18.88)	
≥90	5814 (8.04)	231 (10.14)	30 (5.75)	27 (5.20)	
**Hyperlipidemia**					0.0088
No	67,960 (93.98)	2159 (94.78)	499 (95.59)	502 (96.72)	
Yes	4356 (6.02)	119 (5.22)	23 (4.41)	17 (3.28)	
**Stroke**					0.0434
No	71,970 (99.52)	2273 (99.78)	552 (100.00)	519 (100.00)	
Yes	346 (98.58)	5 (0.22)	0 (0.00)	0 (0.00)	
**Education**					<0.0001
University and above	44,462 (61.48)	1718 (75.42)	428 (81.99)	450 (86.71)	
Senior high	20,483 (28.32)	473 (20.76)	84 (16.09)	58 (11.18)	
Junior high	4539 (6.28)	57 (2.50)	6 (1.15)	10 (1.93)	
Elementary and below	2832 (3.92)	30 (1.32)	4 (0.77)	1 (0.19)	
**Marital status**					<0.0001
Married	51,518 (71.24)	1668 (73.22)	276 (52.87)	285 (54.91)	
Single	11,972 (16.56)	449 (19.71)	198 (37.93)	202 (38.92)	
Divorced	6433 (8.90)	131 (5.75)	38 (7.28)	28 (5.39)	
Widowed	2393 (3.31)	30 (1.32)	10 (1.92)	4 (0.77)	

Values are presented as mean ± standard deviation or *n* (%). *p < 0.05.

HDL-C: high-density lipoprotein cholesterol; LDL-C: low-density lipoprotein cholesterol; BMI: body mass index; WHR, waist-to-hip ratio; ALT: alanine aminotransferase; AST: aspartate aminotransferase; SBP: systolic blood pressure; DBP: diastolic blood pressure.

Table 2 presents two models that demonstrate the associations of HDL-C with jogging (Model 1) and weight training (Model 2). After adjusting for confounders, jogging and weight training (compared to no exercise) were significantly associated with higher levels of HDL-C (β; *p* = 2.5472; <0.0001 for jogging and 2.6234; 0.032 for weight training).

**Table 2.** Association of high-density lipoprotein cholesterol with jogging (Model 1) and weight training (Model 2).

	Model 1		Model 2	
	*β*	*p*-Value	*β*	*p*-Value
**Exercise type (Ref: no exercise)**				
Jogging	2.5472	<0.0001	-	-
Weight training	-	-	2.6234	<0.0001
**Sex (Ref: female)**				
Male	−7.8960	<0.0001	−7.9130	<0.0001
**Age (Ref: 30-45)**				
46–60	0.7863	<0.0001	0.7915	<0.0001
≥61	0.9666	<0.0001	0.9801	<0.0001
**Creatinine (Ref: <1.4)**				
≥1.4	−1.1513	0.0334	−1.2279	0.0242
**Uric acid (Ref: ≤6)**				
>6	−1.5240	<0.0001	−1.5231	<0.0001
**Total cholesterol (Ref: <200)**				
≥200	11.1380	<0.0001	11.0619	<0.0001
**Triglycerides (Ref: <150)**				
≥150	−9.7095	<0.0001	−9.6818	<0.0001
**LDL-C (Ref: <130)**				
≥130	−6.9322	<0.0001	−6.8877	<0.0001
**BMI (Ref: normal)**				
Underweight	4.2380	<0.0001	4.1883	<0.0001
Overweight	−2.7285	<0.0001	−2.6684	<0.0001
Obese	−3.3714	<0.0001	−3.3256	<0.0001
**WHR (Ref: male <0.9; female <0.8)**				
Male ≥0.9; female ≥0.8	−2.1074	<0.0001	−2.1163	<0.0001
**Cigarette smoking (Ref: never)**				
Quit	0.0567	0.6881	0.0344	0.8120
Current	−1.9929	<0.0001	−1.9858	<0.0001
**Alcohol drinking (Ref: never)**				
Quit	0.0487	0.8461	0.1338	0.6001
Current	4.7066	<0.0001	4.6719	<0.0001

Adjusted for betel nut chewing, AST, ALT, waist circumference, body fat, diabetes, fasting blood glucose, hypertension, SBP, DBP, hyperlipidemia, stroke, education, and marital status.

Ref: reference; LDL-C: low-density lipoprotein cholesterol; BMI: body mass index; WHR: waist-to-hip ratio; ALT: alanine aminotransferase; AST: aspartate aminotransferase; SBP: systolic blood pressure; DBP: diastolic blood pressure.

Table 3 presents the association between HDL-C and exercise, with both jogging and weight training included in the same model. HDL-C was positively associated with jogging and weight training (β = 2.5478, *p* < 0.0001 and β = 2.6230, *p* < 0.0001, respectively). It was also positively associated with TC (β = 11.1363, *p* < 0.0001), underweight (β = 4.2429, *p* < 0.0001), former smoking (β = 0.0669, *p* = 0.6348), and current drinking (β = 4.7049, *p* < 0.0001). However, it was negatively associated with the male sex (β = −7.9005, *p* < 0.0001), triglycerides (β = −9.7032, *p* < 0.0001), LDL-C (β = −6.9361, *p* < 0.0001), overweight (β = −2.7294, *p* < 0.0001), obesity (β = −3.3728, *p* < 0.0001), WHR (β = −2.1007, *p* < 0.0001), and current smoking (β = −1.9856, *p* < 0.0001).

**Table 3.** Association of high-density lipoprotein cholesterol with jogging and weight training (both exercise types are included in the same model).

	*β*	*p*-Value
**Exercise type (Ref: no exercise)**		
Jogging	2.5478	<0.0001
Weight training	2.6230	<0.0001
**Sex (Ref: female)**		
Male	−7.9005	<0.0001
**Age (Ref: 30-45)**		
46–60	0.7803	<0.0001
≥61	0.9618	<0.0001
**Creatinine (Ref: <1.4)**		
≥1.4	−1.1497	0.0337
**Uric acid (Ref: ≤6)**		
>6	−1.5146	<0.0001
**Total cholesterol (Ref: <200)**		
≥200	11.1363	<0.0001
**Triglycerides (Ref: <150)**		
≥150	−9.7032	<0.0001
**LDL (Ref: <130)**		
≥130	−6.9361	<0.0001
**BMI (Ref: normal)**		
Underweight	4.2429	<0.0001
Overweight	−2.7294	<0.0001
Obese	−3.3728	<0.0001
**WHR (Ref: male <0.9; female <0.8)**		
Male ≥0.9; female ≥0.8	−2.1007	<0.0001
**Cigarette smoking (Ref: never)**		
Quit	0.0669	0.6348
Current	−1.9856	<0.0001
**Alcohol drinking (Ref: never)**		
Quit	0.0300	0.9045
Current	4.7049	<0.0001

Adjusted for betel nut chewing, AST, ALT, waist circumference, body fat, diabetes, fasting blood glucose, hypertension, SBP, DBP, hyperlipidemia, stroke, education, and marital status.

Ref: reference; LDL-C: low-density lipoprotein cholesterol; BMI: body mass index; WHR: waist-to-hip ratio; ALT: alanine aminotransferase; AST: aspartate aminotransferase; SBP: systolic blood pressure; DBP: diastolic blood pressure.

Table 4 presents the association between HDL-C and exercise, with jogging, weight training, and mixed exercise included in the same model. HDL-C was positively associated with jogging, weight training, and mixed exercise compared with no exercise (β = 2.5470, *p* < 0.0001; β = 2.6249, *p* < 0.0001; and β = 3.2177, *p* < 0.0001, respectively). The β value for mixed exercise was higher than that for jogging and weight training (*p* for trend <0.0001). HDL-C was also positively associated with TC (β = 11.1566, *p* < 0.0001), underweight (β = 4.2316, *p* < 0.0001), and current drinking (β = 4.7041, *p* < 0.0001). However, it was negatively associated with the male sex (β = −7.9130, *p* < 0.0001), triglycerides (β = −9.7179, *p* < 0.0001), LDL-C (β = −6.9575, *p* < 0.0001), overweight (β = −2.7284, *p* < 0.0001), obesity (β = −3.3711, *p* = 0.0036), WHR (β = −2.1014, *p* < 0.0001), and current smoking (β = −1.9977, *p* < 0.0001).

**Table 4.** Association of high-density lipoprotein cholesterol with jogging, weight training, and mixed exercise (all the exercise types are included in the same model).

	*β*	*p*-Value
**Exercise type (Ref: no exercise)**		
Jogging	2.5470	<0.0001
Weight training	2.6249	<0.0001
Mixed	3.2177	<0.0001
*p* for trend < 0.0001		
**Sex (Ref: female)**		
Male	−7.9130	<0.0001
**Age (Ref: 30–45)**		
46-60	0.7950	<0.0001
≥61	0.9729	<0.0001
**Creatinine (Ref: <1.4)**		
≥1.4	−1.1459	0.0343
**Uric acid (Ref: ≤6)**		
>6	−1.5264	<0.0001
**Total cholesterol (Ref: <200)**		
≥200	11.1566	<0.0001
**Triglycerides (Ref: <150)**		
≥150	−9.7179	<0.0001
**LDL (Ref: <130)**		
≥130	−6.9575	<0.0001
**BMI (Ref: Normal)**		
Underweight	4.2316	<0.0001
Overweight	−2.7284	<0.0001
Obese	−3.3711	<0.0001
**WHR (Ref: male <0.9; female <0.8)**		
Male≥0.9; female ≥0.8	−2.1014	<0.0001
**Cigarette smoking (Ref: never)**		
Quit	0.0769	0.5824
Current	−1.9977	<0.0001
**Alcohol drinking (Ref: never)**		
Quit	0.0691	0.7815
Current	4.7041	<0.0001

Adjusted for betel nut chewing, AST, ALT, waist circumference, body fat, diabetes, fasting blood glucose, hypertension, SBP, DBP, hyperlipidemia, stroke, education, and marital status.

Ref: reference; LDL-C: low-density lipoprotein cholesterol; BMI: body mass index; WHR: waist-to-hip ratio; ALT: alanine aminotransferase; AST: aspartate aminotransferase; SBP: systolic blood pressure, DBP: diastolic blood pressure.

## 6. Discussion

The present study evaluated the differential effects of jogging exercise, weight training, and mixed exercise on HDL-C levels in Taiwanese adults. The results revealed that engaging in both jogging and weight training (mixed exercise) might be more beneficial to HDL-C than just jogging or weight training alone. These findings are consistent with the findings from previous studies. For instance, a randomized controlled study found significant increases in HDL-C levels in obese women after aerobic exercise interventions [28]. According to a meta-analysis of 25 RCTs, aerobic exercise appears to raise HDL-C levels modestly [37]. Other studies have reported a positive relationship between resistance exercise and HDL [21,22]. In an RCT on obese adolescent boys, both aerobic and resistance exercise lowered abdominal fat and intrahepatic lipid contents [50]. Moreover, in an observational study conducted by Hsu and colleagues, both aerobic and resistance exercise had a positive association with HDL-C levels, with resistance exercise having a stronger association [16]. Intervention and observation studies found that aerobic exercise combined with resistance exercise was associated with higher HDL levels among overweight and obese individuals [14–16]. However, in a randomized trial comparing the effects of aerobic and resistance training regimens on coronary risk factors, only aerobic exercise was significantly associated with higher HDL [51]. In another study involving adolescent girls, aerobic exercise significantly lowered the intrahepatic lipid content and visceral fat, while resistance exercise did not [36]. However, RCTs and meta-analyses of RCTs found no significant changes in HDL before and after aerobic exercise [25,26,30,41]. Another RCT assessing the effect of resistance training on lipid profiles of premenopausal women found no significant differences in HDL before and after the training [23]. In addition, other studies did not find any significant improvements in HDL when the two exercise modalities were combined [43,44].

To our knowledge, no comprehensive study has been conducted on the effects of jogging alone or in combination with weight training on HDL levels. In contrast to our study, a previous study involving 26 women found that weight training and jogging decreased HDL levels. However, the decrease was significant only among individuals who jogged [35].

There is no clear mechanism underlying the positive relationship between HDL and exercise. In part, this may be due to the modulation of hepatic metabolism by exercise, which enhances the expression and function of liver enzymes involved in cholesterol transport. For instance, exercise has been associated with a higher expression of ATP-binding cassette transporter A-1 in the liver [18]. This enzyme plays a very important role in HDL formation and reverse cholesterol transport and is highly correlated with HDL-C levels [19,20]. Exercise has also been associated with higher levels of the transcription factor, liver X receptor [20], which upregulates the ABCA1 protein [34,52]. Based on this concept, our results suggest that regular mixed exercise (jogging and weight training) may improve HDL-C levels, which could in turn increase reverse cholesterol transport and insulin sensitivity, reducing CVD and metabolic syndrome risk.

The current study has several limitations. First, the cross-sectional design precluded the determination of a causal relationship between exercise type and HDL-C levels. Therefore, future studies should adopt experimental designs to evaluate causality. Second, subjects belonging to the exercise groups reported performing exercise at least three times per week for ≥30 min per session, but interindividual variation did not exist in the exercise durations or intensities. The current study included only a subset of Taiwanese (healthy adults aged 30–70 years) who volunteered to be enrolled in the TWB project. The findings might not be generalized to the entire Taiwanese population, Southeast Asians, and individuals from other areas like the United States and Austria. Next, the differences in sample sizes may have affected statistical comparisons between our study groups. Finally, the results of our study showing a positive association between HDL and exercise may have been confounded by other factors, such as lower weight, lower WHR, and others; therefore, these factors should be taken into account in the future research.

## 7. Conclusion

In this study involving Taiwanese adults aged 30–70 years, jogging and weight training were independently associated with increased levels of HDL-C. The increase in HDL-C concentrations appeared greater among individuals who engaged in both jogging and weight training. Different exercise types should be considered in future prospective intervention studies such as RCTs to establish a causal relationship between exercise types and HDL-C.

## Data Availability

The datasets generated and/or analyzed during the current study are available from the corresponding author on reasonable request.
